# *FAN1* Deletion Variant in Basenji Dogs with Fanconi Syndrome

**DOI:** 10.3390/genes15111469

**Published:** 2024-11-14

**Authors:** Fabiana H. G. Farias, Tendai Mhlanga-Mutangadura, Juyuan Guo, Liz Hansen, Gary S. Johnson, Martin L. Katz

**Affiliations:** 1 Canine Genetics Laboratory, Department of Veterinary Pathobiology, College of Veterinary Medicine, University of Missouri, Columbia, MO 65211, USA; ffboston2021@gmail.com (F.H.G.F.); tendai@missouri.edu (T.M.-M.); guoj@missouri.edu (J.G.); hansenl@missouri.edu (L.H.);; 2 Neurodegenerative Diseases Research Laboratory, Department of Ophthalmology, University of Missouri, Columbia, MO 65212, USA

**Keywords:** kidney, hereditary disorder, DNA repair, whole genome sequencing, toxins

## Abstract

**Background:** Fanconi syndrome is a disorder of renal proximal tubule transport characterized by metabolic acidosis, amino aciduria, glucosuria, and phosphaturia. There are acquired and hereditary forms of this disorder. A late-onset form of Fanconi syndrome in Basenjis was first described in 1976 and is now recognized as an inherited disease in these dogs. In part because of the late onset of disease signs, the disorder has not been eradicated from the breed by selective mating. A study was therefore undertaken to identify the molecular genetic basis of the disease so that dogs could be screened prior to breeding in order to avoid generating affected offspring. **Methods:** Linkage analysis within a large family of Basenjis that included both affected and unaffected individuals was performed to localize the causative variant within the genome. Significant linkage was identified between chromosome 3 (CFA3) makers and the disease phenotype. Fine mapping restricted the region to a 2.7 Mb section of CFA3. A whole genome sequence of a Basenji affected with Fanconi syndrome was generated, and the sequence data were examined for the presence of potentially deleterious homozygous variants within the mapped region. **Results:** A homozygous 317 bp deletion was identified in the last exon of *FAN1* of the proband. 78 Basenjis of known disease status were genotyped for the deletion variant. Among these dogs, there was almost complete concordance between genotype and phenotype. The only exception was one dog that was homozygous for the deletion variant but did not exhibit signs of Fanconi syndrome. **Conclusions:** These data indicate that the disorder is very likely the result of FAN1 deficiency. The mechanism by which this deficiency causes the disease signs remains to be elucidated. FAN1 has endonuclease and exonuclease activity that catalyzes incisions in regions of double-stranded DNA containing interstrand crosslinks. FAN1 inactivation may cause Fanconi syndrome in Basenjis by sensitization of kidney proximal tubule cells to toxin-mediated DNA crosslinking, resulting in the accumulation of genomic and mitochondrial DNA damage in the kidney. Differential exposure to environmental toxins that promote DNA crosslink formation may explain the wide age-at-onset variability for the disorder in Basenjis.

## 1. Introduction

Fanconi syndrome (FS) is characterized by excessive frequent urination (polyuria), excessive thirst (polydipsia), bone pain, and muscle weakness [1,2,3]. The disorder was first described by Dr. Guido Fanconi in 1936 [4]. FS results from impaired function of the proximal renal tubular epithelial cells, leading to urinary leakage of phosphate, glucose, uric acid, amino acids, low-molecular-weight polypeptides, and other small molecules, and to proximal renal tubular acidosis [5]. While inherited forms of isolated human FS have been described [6,7], hereditary human FS usually occurs as a component of multisystem disorders such as mitochondrial cytopathies [8], Dent’s disease [9], Lowe’s syndrome [10] and cystinosis [11]. FS can also result from the toxic effects of certain drugs or heavy metals on the proximal tubules of the kidneys in individuals with no known genetic risk factors [12,13,14,15,16].

Canine FS was first reported in Basenjis by Easley and Breitschwerdt in 1976 [17]. Additional reports of FS in this dog breed have followed [18,19,20,21,22,23,24,25]. Based on these reports, the disease in Basenjis appears to be inherited as an autosomal recessive trait. Typically, the first signs of FS in Basenjis are polydipsia, polyuria, weight loss, and poor hair coat [25]. In most Basenjis, the age of onset is between 4 and 7 years of age, and the lifespan of affected dogs is between 11 and 12 years of age if they have been maintained with dietary management [25]. There have been reports of sporadic FS in other dog breeds, some of which have been linked to dietary factors or heavy metals [25,26,27,28,29,30,31,32,33,34,35,36,37], although these cases do not exhibit some of the laboratory findings characteristic of Basenji FS and may present with additional signs not seen in the Basenji disease.

Among Basenjis, the prevalence of FS has been estimated to be about 10% [24]. Variants in a number of genes have been associated with human FS-like disorders [38,39,40,41,42,43,44,45,46,47,48,49,50,51,52,53], but the molecular genetic basis of the canine disorder has not been determined. Because of the late and variable onset of disease signs, it has not been possible to eradicate the disorder from the breed by identifying dogs that carry the genetic risk variant based on disease phenotype. Identifying this variant would enable breeders to screen dogs prior to mating so that they could avoid propagating the disease. Genetic linkage and whole genome sequence studies were therefore undertaken to identify the cause of the Basenji disease.

## 2. Materials and Methods

**Figure 1:**
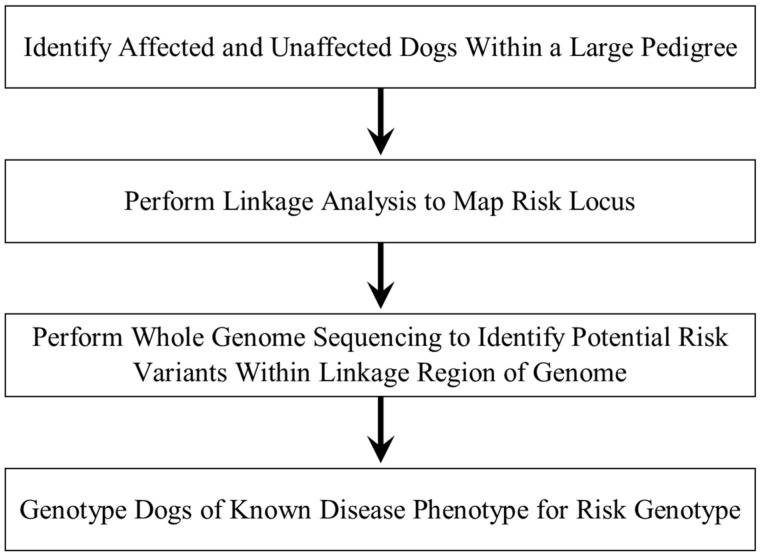
Strategy for identifying the DNA sequence variant responsible for FS in Basenjis.

In order to identify the potential DNA sequence variant responsible for FS in Basenjis, the disease phenotypes of dogs within a large pedigree were ascertained. DNA samples from these dogs were used to perform linkage mapping of the disease locus followed by whole genome sequence analysis to identify potential risk variants within the mapped region. All of the dogs within the pedigree were then genotyped for a candidate variant to assess concordance between phenotype and genotype. This strategy is summarized in Figure 1.

Genomic DNA was isolated from blood leukocytes as described previously [54]. Genetic mapping of the FS locus was performed using a 325 microsatellite marker canine linkage map to genotype a 59-member family of Basenjis, including 22 afflicted with FS. Linkage analysis of the disease locus genotypes inferred from phenotypes under a completely penetrant autosomal recessive model of inheritance and marker loci was performed using Cri-map 2.507 software (http://www.animalgenome.org/bioinfo/resources/manuals/Embnetut/Crimap/ accessed 15 August 2019). In addition to the 11 canine chromosome 3 (CFA3) markers included in the original linkage map panel, we used another 29 CFA3 microsatellite markers (Supplemental Table S1) for fine mapping by haplotype homozygosity in FS-affected Basenjis but not in Basenjis with normal renal function.

For whole-genome sequencing, a 300 bp paired-end library was prepared with the Illumina TruSeq sample preparation kit and DNA from a single FS-affected Basenji. The library was sequenced in a 2 × 120 cycle run in 2 lanes of a flow cell from an Illumina Genome Analyzer II and in a 2 × 100 cycle run in 1 lane from an Illumina HiSeq 2000 (Illumina Inc., San Diego, CA, USA). We used the same procedures to obtain whole genome sequences from 3 dogs of other breeds in unrelated projects. These dogs were not affected by FS, and consequently, the produced sequences served as controls for this project. The control dogs suffered from neurological disorders and had not exhibited any signs of FS. Reads from all sequences were aligned to the canine reference sequence build v2.1 using NextGENe v2.15 software. To identify candidate pathogenic mutations, we performed an exon-by-exon inspection of all genes within the fine-mapped disease-associated region for potentially deleterious mutations. We also evaluated sequence gaps within the mapped region for the likelihood that they represented disease-related genomic DNA deletions. For this analysis, we generated NextGENe Expression reports with 100 bp windows to identify coverage gaps that were unique to the FS-affected Basenji and that included exonic DNA. We amplified across the single gap fulfilling these criteria with PCR primers 5′-ATATATAGTAGAGCAGTATCAGT-3′ and 5′-ATTTCCTAAAATGGCCAC-3′ and confirmed the identity of the resulting amplicons by automated Sanger sequencer (3730xl; Applied Biosystems, Waltham, MA, USA). DNA samples from individual dogs were genotyped for the deletion allele with the same primers used to validate the deletion. These primers produce amplicons of 480 bp for the wild-type allele and 163 bp for the mutant allele. Amplicon sizes were determined with a microcapillary system (QIAxcel, Qiagen N.V., Venlo, The Netherlands). An RNeasy kit (Qiagen) was used to extract total RNA from the kidney of two Dachshunds obtained after euthanasia for an unrelated health problem. Additionally, total RNA was extracted from the white blood cells and serum of FS-affected and FS-unaffected dogs with the PAXgene Blood RNA kit (Qiagen). RT-PCR amplifications were performed with a GeneAmp^®^EZ *rTth* RNA PCT kit (Applied Biosystems) using the primer pairs in Table 1. We also performed 3′-RACE amplifications with the Invitrogen 3′ RACE System with two specific primers from exon 13: 5′-GCTGTGGACTTCCGACACT-3′ for the first amplification and 5′-CTCCCAGAGTCATCGTGTT-3′ for the nested amplification. The identities of the resulting amplicons were verified by automated Sanger sequencing.

## 3. Results

**Figure 2:**
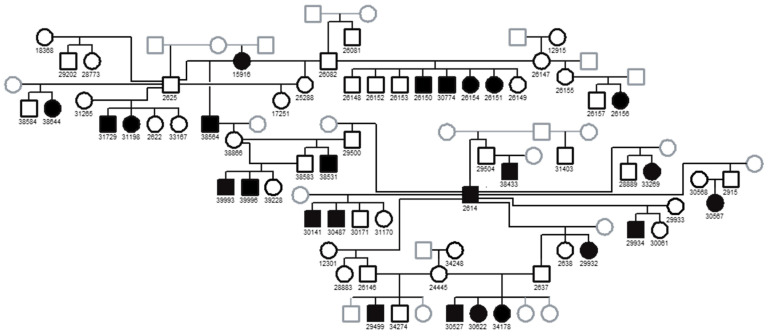
Pedigree of the Basenji family used for linkage mapping of the FS locus. Filled black squares: affected males; filled black circles: affected females; open black squares: unaffected males; open black circles: unaffected females; gray open squares, males of unknown phenotype; gray open circles: females of unknown phenotype.

**Figure 3:**
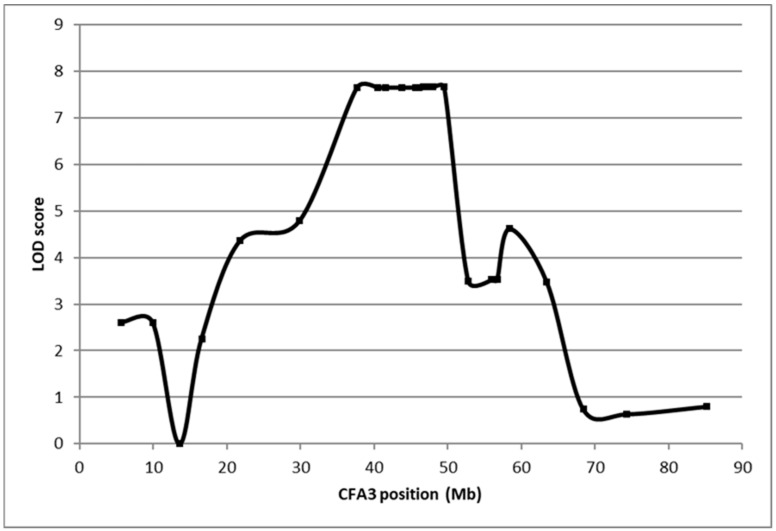
Plot of linkage results of the Fanconi syndrome Basenji pedigree (22 cases and 37 controls).

Linkage analysis was performed by genotyping DNA from a Basenji family consisting of 22 FS cases and 37 FS-unaffected controls for 325 genome-wide microsatellite loci (Figure 2). The strongest associations with inferred genotypes for the FS locus and marker loci occurred on CFA3 (Figure 3). Fine mapping in 86 FS-affected Basenjis revealed that these dogs were all homozygous for the same 6-marker haplotype flanked by recombinant markers at 40,537,065 bp and 43,218,050 bp, and that none of 11 aged Basenjis with normal renal function were homozygous for this haplotype. This analysis defined a 2.7 Mb target region of CFA3 as harboring the FS locus, which contained 11 annotated genes.

**Figure 4:**
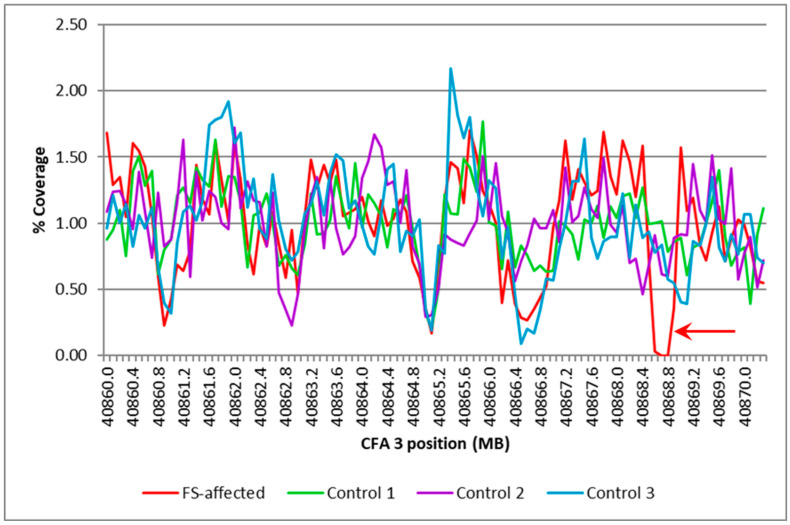
Comparison of percent coverage in a part of the CFA3 target region between an FS-affected dog and three controls. The region represented in this graph starts at 40,860,000 to 40,870,300 bp of CFA3 in intervals of 100 bp. The percent coverage is the average coverage of the interval normalized for the average genome coverage for the individual dog. The arrow indicates the gap in coverage that was unique to the affected Basenji.

**Figure 5:**
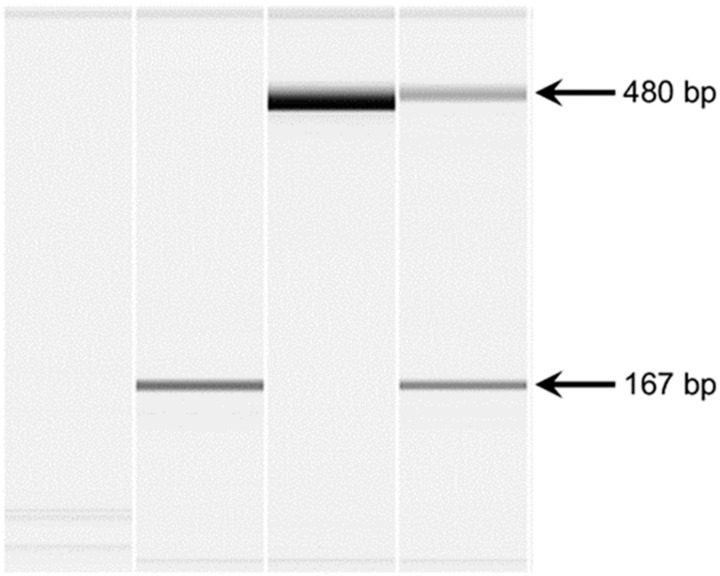
Microcapillary electrophoretograms of PCR amplicons produced with primers spanning the gap in the *FAN1*. Lane 1 represents a negative control. PCR was performed with DNA from an FS-affected dog (2) and two FS-unaffected dogs (lanes 3 and 4). The FS-affected dog produced a 167 bp amplicon, which is smaller than the expected band. One of the FS-unaffected dogs (3) produced the expected band, and the other dog (lane 4) produced the expected band and the deletion allele band.

**Figure 6:**
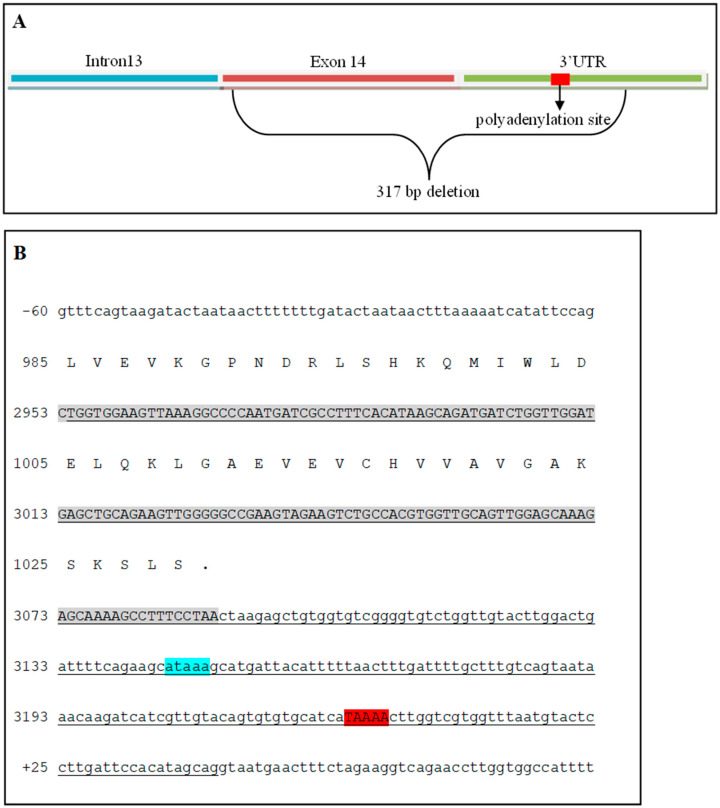
The deletion boundaries are represented by an illustration and genomic sequences of the *FAN1*. (**A**) Illustration of the 3′ end of the *FAN1* gene. The deletion starts after the first base of exon 14 and goes into the 3′ UTR past the primary polyadenylation site. (**B**) Sequences for the end of intron 13, exon 14, and the 3′UTR. Gray-shaded sequences correspond to exon 14, blue-shaded represents the potential poly signal, and red-shaded is the polyadenylation site. The 317 deleted bases are underlined.

The whole-genome sequence reads from the Illumina Genome Analyzer II and Hiseq 2000 were combined and aligned to the canine genome reference to produce an aligned sequence with 12.7-fold average coverage. Exon-by-exon inspection of the sequences for the 143 annotated exons in the 11 genes within the FS region failed to reveal any sequence variants likely to alter the function of the gene products. This inspection also revealed gaps in the aligned sequence that overlapped part or all of the 11 exons from within the FS target region. Comparisons of the depth of coverage in the WGS of the FS-affected Basenji with those of the WGS from 3 unaffected dogs of other breeds showed similar patterns for all but one of the sequence gaps. The exception was found only in the Basenji sequence in the vicinity of *FAN1* exon 14 (Figure 4). PCR amplification with primers spanning the gap confirmed that a deletion in the genomic DNA of the FS-affected Basenji was responsible for the gap (Figure 5), and re-sequencing the amplicons produced with these primers revealed that 317 bp of exon 14 were deleted starting at the second exon14 nucleotide and extending into the 3′ untranslated region of *FAN1* (Figure 6). In addition, the mutant allele has four nucleotide substitutions within the 12 nucleotides immediately 3′ to the deletion.

**Figure 7:**
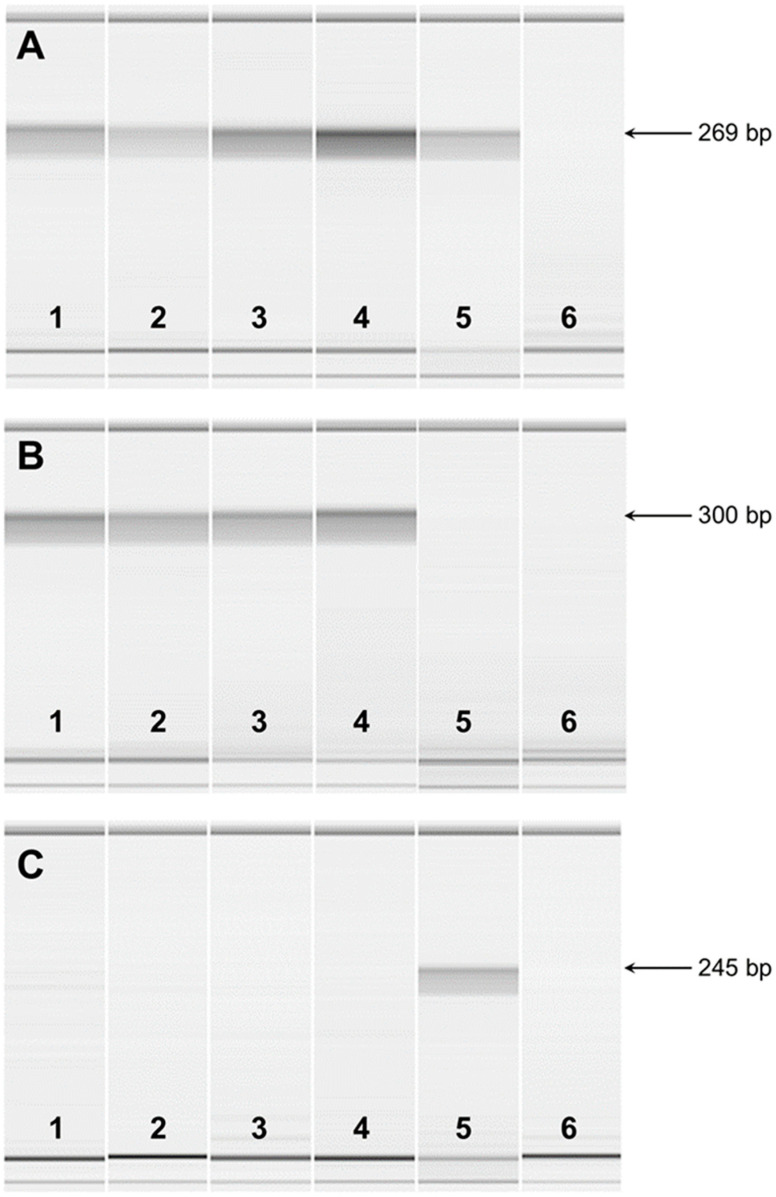
Microcapillary electrophoretograms of RT-PCR amplicons from *FAN1* mRNA. RT-PCR was performed with total RNA extracted from the kidneys of two FS-unaffected dogs (1 and 2), the blood of two FS-unaffected dogs (3 and 4), and one FS-affected dog (5). Lane 6 represents a negative control. (**A**) RT-PCR was performed with primers from exon 5 to exon 7 of the *FAN1* gene. The expected amplicon size was 269 bp. (**B**) RT-PCR was performed with primers from exon 12 to exon 14 of the *FAN1* gene. The expected amplicon size was 300 bp. (**C**) RT-PCR was performed with primers from exon 12 to intron 13 of the *FAN1* gene. The expected amplicon size was 245 bp.

RT-PCR was used to analyze the *FAN1* transcripts present in the total RNA from the kidneys of 2 unaffected dogs and from the blood of FS-affected and unaffected dogs. All of the RNA preparations produced similar RT-PCR amplicons with primers designed from exon 5 and 7 sequences. Microcapillary electrophoretograms of RT-PCR amplicons demonstrated expression in all samples, indicating that the mutant transcript is transcribed (Figure 7A). As expected, RT-PCR with primers designed from exon 12 and from the deleted region of exon 14 produced amplicons with RNA from normal but not affected dogs (Figure 7B). Primers designed from exon 12 and intron 13 produced amplicons with RNA from affected but not normal dogs (Figure 7C), indicating that the deletion causes intron retention in the transcript. RNA samples for both normal and affected dogs failed to produce RT-PCR amplicons with primers designed from exon 13 and sequences immediately 3′ to the deletion, suggesting that the deletion includes the polyadenylation site for normal dogs. This was confirmed with a 3′ RACE experiment, which located the normal polyadenylation site 135 bp past the stop codon and 76 bp past a potential polyA signal. A similar 3′ RACE experiment revealed that the mutant RNA produces a transcript of 577 bp into intron 13, 520 bp past an in-frame termination codon.

We genotyped a cohort of 78 Basenjis of known clinical status for the *FAN1* deletion and found all 32 of the FS-affected Basenjis to be homozygous for the deletion allele. FS-unaffected dogs tested either homozygous wild-type or heterozygous, except for one unaffected dog that tested homozygous for the deletion allele. The deletion allele was highly significantly associated with the FS phenotype (*p* = 4.219 × 10^−21^, Fisher’s exact test 2 × 2). Table 2 summarizes the genotype distribution.

## 4. Discussion

The data from this study indicate that FS in Basenjis is the result of FAN1 deficiency. The homozygous deletion genotype was strongly associated with the FS phenotype, with only one dog that did not exhibit disease signs out of 33 that were homozygous for the deletion variant. In addition, the *FAN1* risk variant was not present in 120 unaffected dogs from 81 different breeds. The whole genome sequence of the proband did not contain homozygous variants in any other genes that have been associated with the type of disease signs exhibited by the affected Basenjis. Although a functional assay of potential FAN1 enzymatic activity was not performed, the predicted translation of the variant transcript suggests a grossly altered protein structure if the transcript is translated (Supplemental Figure S1). *FAN1* variants have been associated with FS-like disorders in human subjects and mice [55,56,57], but this is the first report of *FAN1*-associated FS in dogs.

The mechanism by which deficiency in FAN1 leads to the kidney pathology associated with FS remains to be fully elucidated. *FAN1* was first named *KIAA1018* by Nagase et al. [58], who screened brain cDNA libraries for unidentified genes. They determined that *KIAA1018* was expressed at similar levels in multiple tissues. Alonso et al. [59] proposed that *FAN1* was part of the myotubularin gene family of tyrosine phosphatases. They proposed a new genomic designation, *MTMR15*, and predicted that the encoded protein was a catalytically inactive member of the inactive MTMR family of protein tyrosine phosphatases. The inactive MTMRs have been reported to act as regulatory units for active members of the group [60]. However, no studies have been reported that directly support the hypothesis that FAN1 (MTMR15/KIAA1018) is involved in the regulation of the enzymatically active MTMRs.

Unlike the MTMR proteins, FAN1 has a ubiquitin-binding domain at the N-terminus and at its C-terminus a domain with homology to bacterial and phage endonucleases, which suggests that this protein may contribute to the maintenance of genome stability [61]. FAN1 has been identified as one of the proteins involved in DNA inter-strand crosslink repair [62,63,64,65,66,67]. The disease Fanconi anemia is a recessive disorder characterized by genome instability, impaired repair of DNA crosslink damage, developmental abnormalities, early-onset bone marrow failure, and a predisposition to cancer [68,69]. Variants in more than 20 genes, including *FAN1*, have been associated with Fanconi anemia [69,70,71,72,73]. The name FAN1 (Fanconi anemia-associated nuclease 1) has been proposed because this protein interacts with Fanconi anemia pathway proteins [62,63,64,65,72]. When DNA inter-strand crosslinks occur, FAN1 is recruited to the lesion sites through an interaction between its ubiquitin-binding domain and the ubiquitylated complex of the Fanconi anemia pathway [62,63,64,65,72]. However, it appears that FAN1 may also mediate DNA repair independent of other proteins in this pathway [66,74]. The deleted region encodes a conserved segment of the nuclease domain, which is likely to obliterate FAN1 nuclease activity (Supplemental Figure S2) and thus its role in DNA repair. Based on the evidence that FAN1 is involved in repairing DNA inter-strand crosslinks, the proximal renal tubule pathology associated with FS may be the result of the accumulation of these crosslinks in renal tubule epithelial cells. 

Support for this hypothesis comes from the finding that FAN1 deficiency sensitizes cultured cells to reagents that cause targeted DNA damage. This sensitivity can be rescued by transfection with wild-type *FAN1* but not by variant constructs containing point mutations in the ubiquitin-binding or endonuclease domains, which indicates that both domains are involved in DNA repair [62,63,64,65]. The nuclease domain of FAN1 has both 5′exonuclease activity and endonuclease activities that are key characteristics of DNA repair proteins [62,63,64,65]. 

The fact that the most apparent pathology associated with FS occurs in the proximal tubules of the kidneys suggests that this tissue may be particularly susceptible to DNA damage. Factors that promote DNA damage in this tissue may contribute to the development of FS. Consistent with this hypothesis is the fact that acquired forms of FS have been associated with heavy-metal exposure and toxicoses from drugs such as cisplatin that promote DNA damage [7,14,28,29,75,76,77,78,79]. Heavy metals, such as cadmium, may be present in plants and sea food because of contaminated soils and water [75]. Chronic environmental exposure can result in cadmium accumulation to toxic levels that cause kidney disease [75]. Cadmium toxicity can cause DNA damage, including double- and single-stranded breaks [80]. The kidney is particularly susceptible to cadmium and other heavy metal toxicities. Approximately 50% of the accumulated dose of cadmium is stored in the kidney [75]. In mitochondria, cadmium inhibits the respiratory chain, resulting in the generation of reactive oxygen species [81]. This leads to mitochondrial disruption with the release of cytochrome c, resulting in caspase activation causing cell death by apoptosis [82,83]. 

We propose that FAN1 inactivation causes FS in Basenjis by hyper-sensitization of the proximal tubule cells to toxins that mediate DNA damage, including heavy metals such as cadmium. Since FAN1 has been reported to be involved in DNA repair and the knockdown of *FAN1* sensitizes cells to DNA crosslinking agents [62,63,64,65], we predict that environmental and dietary exposure to toxins that promote DNA damage leads to the accumulation of both genomic and mitochondrial DNA damage in the kidney. Differential exposure to environmental toxins may explain the wide age-at-onset variability for FS in Basenjis. It is possible that the variability in age-at-onset and disease severity may also be due in part to genetic factors that modify the disease risk associated with the *FAN1* variant. It would be difficult to identify any such potential genetic modifiers without controlling for potential environmental risk factors.

The key finding of this study is that there is almost complete concordance between *FAN1* genotype and phenotype, indicating that by avoiding breeding dogs that carry the deletion allele, it should be possible to eradicate FS from the breed. Although environmental factors are likely to modify the disease risk among dogs that are homozygous for the deletion allele, none of the dogs that were heterozygous or homozygous for the reference *FAN1* allele developed FS. This indicates that FAN1 is completely protective against any environmental factors, such as heavy metal exposure, that can contribute to FS development.

## Figures and Tables

**Table 1:** RT-PCR primer sequences for *FAN1* mRNA.

Target	Forward Primer Sequence/Reverse Primer Sequence	Amplicon Size (bp)
exon 5 to 7	CCTAGGTACACCATCAATCGGAA/ACAGTCCGAGACAAAATCCTT	269
exon 12 to exon 14	CAGGCCCAGGAAGGCAGA/CACGTGGCAGACTTCTACTTCGG	300
exon 12 to intron 13	CAGGCCCAGGAAGGCAGA/AACACAATTATCAGAGAAAAAGCGT	245
exon 13 to 3′UTR	CTGGCTGTGGACTTCCGACA/CTTAACTGGAAACATTGGGTGTG	244

**Table 2:** Distribution of genotypes among healthy and FS-affected Basenjis.

Phenotype	Del/Del	Genotypes Del/Wt	Wt/Wt	Total
FS-affected	32	0	0	32
FS-unaffected	1	33	12	46
Total	33	33	12	78

## Data Availability

WGS DNA sequence data for the Basenji included in this study have been archived and deposited in the NCBI Sequence Read Archive as BioSample SAMN04196845.

## Supplementary Materials

The following supporting information can be downloaded at: https://www.mdpi.com/article/10.3390/genes15111469/s1, Figure S1: Wild-type and mutant cDNA sequences and mRNA illustration of *FAN1*; Figure S2: Aligned amino acid sequences for the C-terminal of the FAN1 protein; Table S1: Chromosome 3 microsatellite markers used for disease locus mapping.
