# Supplementary material for: FAN1 Deletion Variant in Basenji Dogs with Fanconi Syndrome
Source: Genes (Basel). 2024 Nov 14;15(11):1469. doi: 10.3390/genes15111469 (PMC11593659; doi:10.3390/genes15111469)
Supplement: Supplementary file 1 [file genes-15-01469-s001.zip › genes-3302692-supplementary.pdf]

Supplemental Table S1. Chromosome 3 microsatellite markers used for disease locus mapping.

Primer	Dye	5'-Primer Sequence-3'	Amplicon Size (bp)
UMC0408U	VIC	AAGCTAAAGCAACTAGGCAATGA	196
UMC0408L		TGATAGGCTCCTTTCTCTGCATACTG	
UMC0414U	6FAM	CAGTATTGCCCATCTCGGTGAA	273
UMC0414L		GGACCAGAGCGGATGCAAG	
UMC0415U	VIC	TCAGGTCCTTCAGATGGGTCAATC	159
UMC0415L		ACGTGGAAGATAGTGAACTTTAGCA	
UMC0416U	NED	ATAGATTCTTCCCCTTTATAGACAA	223
UMC0416L		AGGTCTGGATCGGGTTATAGAG	
UMC0417U	6FAM	TCTTTCAATTCCATCCTGGTATGT	203
UMC0417L		AACTATAGCATGTGCAATGAGTGAGTGAT	
UMC0418U	PET	TGTGAGTGAGGTCAGAGGCAATGT	221
UMC0418L		CCTTTCTCTCGGATACAATCCTGT	
UMC0419U	VIC	GGGCTTTCTGAGGGATTGAAG	224
UMC0419L		GAACTGTTCTTTCCAAGGGTTAGG	
UMC0420U	NED	AGCCAGAATCTGTAGGTTGCCA	195
UMC0420L		TGCTCCAGCCTTGAAACGG	
UMC0421U	6FAM	CAAAATCTCATCCCTGGTCATCTAA	190
UMC0421L		CCTGTTCACACTTGCCTTTACTCTT	
UMC0422U	PET	GCTCTTGCCCCAGATCACT	104
UMC0422L		GCCTTAATGACTACCCGATGAC	
UMC0423U	6FAM	GGGAAGAAATAAAACTGGAACCAACT	167
UMC0423L		CATACATATGCATGTGCCTCAATTTACA	
UMC0424U	NED	AAACTTGATCCTTCAATATAGCTGG	155
UMC0424L		GTCTTAATGGTGGCATAAAGGG	
UMC0448U	PET	CTATTTGAGGTCTTTTGTGGTCCTATAC	195
UMC0448L		CATGCTCATGGATCAAAACAATACTGTTA	
UMC0449U	6FAM	GTGAAATTTGGGAAACATCTGGCTATA	197
UMC0449L		CGGAATGAGCACCAGCTAAAGA	
UMC0476U	NED	CACATCTTTCCCTGTCTTCAAGGT	185
UMC0476L		CTGCATTCCTCATTACTGTTCATTCC	
UMC0478U	6FAM	ATCGGCACTAAGATGGACTGTC	212
UMC0478L		AGAGGAGAAATGAATGGGTGACTAGC	
UMC0479U	VIC	TTCAGGGACCCGAGGAT	220
UMC0479L		GTTTTATTCTTCCACCCATTGC	
UMC0480U	6FAM	CCAGGCCTCCTTCACGTTCAG	109
UMC0480L		CCCCCTAATCTCACACAACCAG	
UMC0483U		GGGGTAGCCAGCTCTCAACA	130
UMC0483L	6FAM	CCGTCTCCGAAGCTCCGTGT	
UMC0495U		CCCTACTCCTCCTCCATCTTAACTCTT	162
UMC0495L	VIC	TTCTATCTGCCTCTAGGACTTGTGGTA	
UMC0496U	PET	AAACTAGGAGAGAAAGGTGTGCCATC	146
UMC0496L		CCGGAACCTCGTTTTTGGTG	
UMC0497U	NED	TTTACCAAACTAATTCTGTGTGCAAGA	177
UMC0497L		ACACTAGATTCTCTCCCTTGTTTTTAGTAT	
UMC0498U	6FAM	CAAACTAATTCTGTGTGCAAGACGTAACAT	220
UMC0498L		GCCACCATATAGTCTACTACAGAAGTCATT	
UMC0502U	6FAM	ACTTTCTATTAACTTAAAACCCGTGTA	176
UMC0502L		ATGAGGACTTCTATTTTCCCAGATCCA	
UMC0503U	VIC	TATTTGCAGGCAAGAACTTGATTTAC	117
UMC0503L		ATGGAAAGTCCCAGAGTCTTGCAG	
UMC0504U	PET	GGCTGTTATTTGCAGGCAAGAA	177
UMC0504L		GAAAGTGGCAGCTCTGAGAGAGGT	
UMC0505U	NED	GGTGTCTGGCTTCTATAATTTAGCAT	193
UMC0505U		GCCCAAGGTCAAGTCTTCAGTT	
UMC0506U	VIC	AGATCTCCAATTGATTTCATAGGTGTTT	183
UMC0506L		CCTTCTCCGTGTCTGCTGATACACA	
UMC0507U	VIC	CCCCTACTCCTCCTCCATCTTAAC	218
UMC0507L		TAAGTAGTATTACCAACTGGGCTA	

A

```
 921 Q  E  G  R  V  A  S  I  V  S  W  D  R  F  A  S  L  Q  Q  A
2761 CAGGAAGGCAGAGTGGCTTCCATTGTCAGCTGGGATCGCTTTGCTTCTCTTCAGCAAGCT
 941 Q  D  L  V  S  C  L  G  G  P  V  L  S  G  V  C  R  R  L  A
2821 CAGGATCTTGTTTCCTGCTTGGGCGGCCCTGTCCTCAGTGGTGTGTGCAGGCGCCTGGCT
 961 V  D  F  R  H  C  R  G  G  L  P  D  L  V  V  W  N  S  Q  S
2881 GTGGACTTCCGACACTGCCGAGGTGGCCTCCCGGACCTGGTGGTGTGGAACTCCCAGAGT
 981 H  R  V  K  L  V  E  V  K  G  P  N  D  R  L  S  H  K  Q  M
2941 CATCGTGTTAAGCTGGTGGAAGTTAAAGGCCCCAATGATCGCCTTTCACATAAGCAGATG
1001 I  W  L  D  E  L  Q  K  L  G  A  E  V  E  V  C  H  V  V  A
3001 ATCTGGTTGGATGAGCTGCAGAAGTTGGGGGCCGAAGTAGAAGTCTGCCACGTGGTTGCA
1021 V  G  A  K  S  K  S  L  S  .
3061 GTTGGAGCAAAGAGCAAAAGCCTTTCCTAActaagagctgtggtgtcggggtgtctggt
3121 tgtacttggactgattttcagaagcataaagcatgattacatttttaactttgattttg
3161 ctttgtcagtaataaacaagatcatcgttgtacagtgtgtgcatcatAAAAAAAAAAAA
```

B

```
 921 Q  E  G  R  V  A  S  I  V  S  W  D  R  F  A  S  L  Q  Q  A
2761 CAGGAAGGCAGAGTGGCTTCCATTGTCAGCTGGGATCGCTTTGCTTCTCTTCAGCAAGCT
 941 Q  D  L  V  S  C  L  G  G  P  V  L  S  G  V  C  R  R  L  A
2821 CAGGATCTTGTTTCCTGCTTGGGCGGCCCTGTCCTCAGTGGTGTGTGCAGGCGCCTGGCT
 961 V  D  F  R  H  C  R  G  G  L  P  D  L  V  V  W  N  S  Q  S
2881 GTGGACTTCCGACACTGCCGAGGTGGCCTCCCGGACCTGGTGGTGTGGAACTCCCAGAGT
 981 H  R  V  K  V  S  C  T  R  K  E  N  A  F  S  L  I  I  V  L
2941 CATCGTGTTAAGGTCAGCTGCACCAGAAAGGAGAACGCTTTTTCTCTGATAATTGTGTTA
1001 A  I  .
3001 GCAATTTAAtcaaaggctacaacgtaggcatttcttgcatgactattagtaagattgagt
3061 ccctcgagagctattggattgaaggccacagttccttggtggctgtcggctgggacttcc
3121 tcctcagccccttgcctgtggacctctcctgtccactgtgcagcattccacttgacagct
3181 catttcatcagagcaggcaagtgagaagagccagggtgtgagcaagatggaagccagtcc
3241 ctcacaacctgatctcagaagggacattccttcacttttgctgtattcttctgttagaag
3301 caagtcacagattccagcccccattcccagggaagggatccttcagggtgtgaacagcag
3361 gaggctaaggtgactgggagctgttctctaagctccccgtgagtgccttgttacccgctg
3421 tgacctcagggccacccttgcccgcaccctggtcttactctgctgcctgtcccaggttgg
3481 tttttattattgggctttgagattaaaaacaattctggatacacgtcctAAAAAAAAAAA
```

C

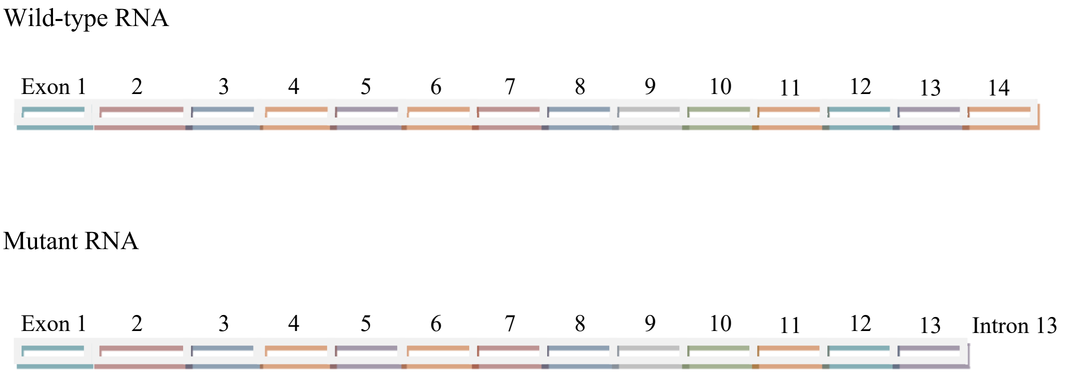

Supplemental Figure S1. Wild-type and mutant cDNA sequences and mRNA illustration of *FAN1*. (A) Wild-type cDNA sequences and protein translation of part of exon 12 (blue), 13 (gray), 14 (blue) and 3’UTR to poly A tail. (B) Mutant cDNA sequences and protein translation of part of exon 12 (blue), 13 (gray) and intron 13 (red) to poly A tail. The mutant transcript is missing 46 amino acids of the end of the protein and has 18 amino acids that are not present in the wild-type protein. (C) Representation of wild-type and mutant RNA. Mutant mRNA has intron 13 in the place of exon 14.

A

FAN1 VRR-NUC DOMAIN

```
Canis lupus familiaris     VDFRHCRGGLPDLVVWNSQSHRVKLVEVKGPNDRLSHKQMIWLDELQKLGAEVEVCHVVAVGAKSKSLS
Homo sapiens               ADFRHCRGGLPDLVVWNSQSRHFKLVEVKGPNDRLSHKQMIWLAELQKLGAEVEVCHVVAVGAKSQSLS
Callithrix jacchus         ADFRHCRGGLPDLVVWNTPSHRCKLVEVKGPSDRLSHKQMIWLDELQKLGAEVEVCHVVAVGAKSQSLS
Pongo abelii               ADFRHCRGGLPDLVVWNSQSHSFKLVEVKGPNDRLSHKQMIWLAELQKLGADVEVCHVVAVGAKSQSLS
Pan troglodytes            ADFRHCRGGLPDLVVWNSQSRHFKLVEVKGPNDRLSHKQMIWLAELQKLGAEVEVCHVVAVGAKSQSLS
Macaca mulatta             ADFRHCRGGLPDLVVWNSQSHRFKLVEVKGPNDRLSHKQMIWLAELQKLGAEVEVCHVVAVGAKSQSLS
Sus scrofa                 VDFRHCRGGLPDLVVWNSQNHRFKLVEVKGPNDRLSHKQMIWLDELRRLGAEVEVCHVVAVGAKSR---
Ailuropoda melanoleuca     ADFRHCRGGLPDLVVWNSQSRHFKLVEVKGPNDRLSHKQMLWLDELQKLGADVEVCHVVAVGAKSKSLT
Equus caballus             VDFRHCRGGLPDLVVWNSQSHRFKLVEVKGPNDRLSHKQMIWLAELQKLGAEVEVCHVVAVGAKSRNLN
Bos taurus                 MDFRHCRGGLPDLVVWSSQSHHVKLVEVKGPHDRLSQKQMIWLDELRRLGADVEVCHVAAVGARSR---
Loxodonta africana         ADFRHCRGGLPDLVVWNSQSHRFKLVEVKGPNDRLSPKQMLWLDELQKLGAEVEVCHVVAVGAKSKGLS
Oryctolagus cuniculus      ADFRHCRGGLPDLVVWSSRSHRFKLVEVKGPNDRLSPKQVIWLDELQRLGAAVEVCHVAAVGAKSQGLS
Mus musculus               ADFRHCRGGLPDLVVWNSQSHHCKLVEVKGPSDRLSCKQMIWLYELQKLGADVEVCHVVAVGAKSKGL-
Rattus norvegicus          ADFRHCRGGLPDLVVWNSQSHHCKLVEVKGPSDRLSCKQMIWLDELQKLGADVEVCHVVAVGAKSKGL-
Heterocephalus glaber      ADFRHCRGGLPDLVVWNSQSRHVKLVEVKGPNDRLSHKQMIWLAELQKLGAEVEVCHVVAVGAKSKGLS
Ornithorhynchus anatinus   TDLRHCRGGLPDLVVWNTQDKRFKLVEVKGPNDRLSHKQMIWLDELQKLGAEVEVCHVVAVGAKGNRLS
Gallus gallus              KDLRHCRGGLPDLVVWSTHSNHFKLVEVKGPNDRLSHKQMIWLSELKKLGAAVEVCHVQAVGARSKRLS
Anolis carolinensis        KDLRHSRGGLPDLVVWRTEDGQYKVVEVKGPNDRLSHKQMLWLAELQELGAAVEVCHVVAEGAKSLHLN
Tetraodon nigroviridis     KDYRHCRAGLPDLVVWNTSENTYKLVEVKGPSDRLSQKQQIWLDELQKLGADVEVCHVVATGARGASL-
```

B

```
Wild-type protein 970 LPDLVVWNSQSHRVKLVEVKGPNDRLSHKQMIWLDELQKLGAEVEVCHVVAVGAKSKSLS*
Mutant protein    970 LPDLVVWNSQSHRVKVSCTRKENAFSLIIVLAI*
```

Supplemental Figure S2. Aligned amino acid sequences for the C-terminal of the FAN1 protein. (A) FAN1 amino acid sequence for the VRR-NUC domain (canine FAN1:p.946-1029) aligned by blastp. Deleted amino acids are marked with black rectangle. (B) Amino acid sequence comparison between wild-type and mutant FAN1.
